# Utility of ^18^F-FDG PET/CT for predicting pathologic complete response in hormone receptor-positive, HER2-negative breast cancer patients receiving neoadjuvant chemotherapy

**DOI:** 10.1186/s12885-020-07505-w

**Published:** 2020-11-16

**Authors:** In Hee Lee, Soo Jung Lee, Jeeyeon Lee, Jin Hyang Jung, Ho Yong Park, Shin Young Jeong, Sang-woo Lee, Yee Soo Chae

**Affiliations:** 1 Department of Oncology/Hematology, Catholic University of Daegu, School of medicine, Daegu, South Korea; 2 Department of Oncology/Hematology, Kyungpook National University Chilgok Hospital, Kyungpook National University, Daegu, South Korea; 3 Department of Breast and Thyroid Surgery, Kyungpook National University Chilgok Hospital, Kyungpook National University, Daegu, South Korea; 4 Department of Nuclear Medicine, Kyungpook National University Chilgok Hospital, Kyungpook National University, Daegu, South Korea

**Keywords:** Breast cancer, Neoadjuvant chemotherapy, Pathologic complete response, SUVmax

## Abstract

**Background:**

Pathologic complete response (pCR) after neoadjuvant chemotherapy (NAC) is a predictor of improved outcomes in breast cancer. In patients with hormone receptor (HR)-positive, human epidermal growth factor receptor 2 (HER2) -negative breast cancer, the response to NAC is variable and mostly limited. This study was an investigation of the predictive relevance of parameters of ^18^F-FDG PET/CT for the pCR to NAC in patients with HR-positive, HER2–negative breast cancer. Methods: AH total of 109 consecutive HR-positive and HER2-negative breast cancer patients who were treated with NAC were enrolled in this prospective cohort study. The relationships between pretreatment ^18^F-FDG PET/CT and clinical outcomes including pathologic response to NAC were evaluated. Results: All patients finished their planned NAC cycles and eight patients (7.3%) achieved pCR. In the receiver operating characteristic (ROC) curve analysis, pSUVmax exhibited high sensitivity and specificity for predicting pCR. Furthermore, multivariate logistic regression analysis revealed pSUVmax as a predictive factor for pCR (hazard ratio = 17.452; 95% CI = 1.847–164.892; *p* = 0.013).

**Conclusion:**

The results of this study suggest that ^18^F-FDG PET/CT pSUVmax is a predictive factor for pCR of HR-positive, HER2-negative breast cancer to NAC.

## Background

Despite remarkable improvement in breast cancer treatment, breast cancer is still one of the most prevalent cancers and the second leading cause of cancer death in women. Therefore, research on new treatment approaches, including neoadjuvant chemotherapy (NAC), is ongoing. NAC was initially introduced for the management of locally advanced or inoperable breast cancers and showed additional advantages, such as down-staging to breast conserving surgery [1–3] and monitoring therapeutic response [4], without significant survival outcomes [5, 6]. For resectable breast cancer, pathological parameters such as tumor size, axillary lymph node involvement, histological grade, hormone receptor (HR) status, and human epidermal growth factor receptor 2 (HER2) status have been used as prognostic factors for survival. Most of these pathological parameters cannot be fully evaluated from small specimens acquired by core needle biopsy in this setting. Several large studies and a meta-analysis revealed that pathologic complete response (pCR) itself predicts survival of patients with aggressive breast cancers, including HER2-positive and triple-negative subtypes [7, 8]. Therefore, the achievement of pCR after NAC has been accepted as a predictive marker of long-term oncologic outcomes and became a surrogate endpoint for prognosis in this setting [9, 10].

Meanwhile, for patients with HR-expressing breast cancer, the most common subtype, achieving pCR is infrequent and did not statistically correlate with survival in a meta-analysis [11]. In this analysis, pCR rate of HR-positive, HER2-negative, low grade breast cancer is 7.5% compared to 33.6% in triple-negative subtype or 50.3% in HER2-positive, HR-negative subtype. The association between pCR and long-term outcomes was strongest in patients with triple-negative breast cancer (Event free survival: HR 0·24, 95% CI 0·18–0·33; OS: 0·16, 0·11–0·25) and in those with HER2-positive, HR-negative tumors (Event free survival: 0·15, 0·09–0·27; OS: 0·08, 0·03, 0·22).

Although, pCR rate is low in HR-positive breast cancer, patients with high grade/HER2-negative or luminal B tumors more frequently achieve pCR, which correlates with better survival and suggests the clinical value of differentiating luminal B from luminal A tumors before NAC [11, 12].

Because subtype determination by molecular assay is expensive [13] and assays of specimens acquired by core needle biopsy do not always correlate with those of whole tumor specimens, new parameters are needed to refine NAC strategies or to estimate survival outcome before NAC.

Fluorine-18 fluorodeoxyglucose positron emission tomography (^18^F-FDG PET/CT) provides quantitative data on the level of metabolic activity by calculating the degree of ^18^F-FDG uptake, represented by the standardized uptake value (SUV), and has shown efficacy in diagnosing, staging, and monitoring various cancers. In breast cancer patients, its efficacy in evaluating chemotherapeutic effects has been reported: a correlation was observed between the intensity of FDG uptake and tumor characteristics such as tumor grade, HR status, and HER-2 status [14–16] and the early metabolic response after one or two courses of NAC was shown to predict pCR, particularly for aggressive subtypes [17, 18]. However, few studies have evaluated the clinical implications of ^18^F-FDG PET/CT in HR-positive- and HER2-negative breast cancer patients. Accordingly, the present study evaluated the utility of SUVmax on PET/CT to predict pCR in breast cancer patients treated with NAC followed by surgery, especially those with the HR-positive and HER2-negative subtype.

## Methods

### Study design

To identify new predictive or prognostic markers for breast cancer from tumor or plasma specimens and functional images such as FDG-PET, we designed a prospective cohort study of breast cancer patients who underwent preoperative chemotherapy at Kyungpook National University Chilgok Hospital (KNUCH), South Korea. The criteria for NAC and study inclusion were tumor size > 2 cm or node positive (stage IIA-IIIC) resectable breast cancer with adequate organ function; patients with cT0 or multiple tumors were excluded from the current study. Patients underwent pretreatment ^18^F-FDG PET/CT combined with conventional radiologic images. NAC regimens were selected based on the presence of lymph node involvement as follows: four cycles of anthracycline + cyclophosphamide (AC4) followed by 4 cycles of docetaxel (T4) for node-positive and 4 cycles of docetaxel + cyclophosphamide (TC4) or AC4 for node-negative tumors. Curative-intent surgery was scheduled to be performed within 6 weeks after the last cycle of NAC and postoperative treatment was adequately done based on the domestic and/or international guidelines. All of the patients received adjuvant endocrine therapy in accordance with ASCO/SABCS guidelines. Follow-up imaging was performed semi-yearly for the initial 3 years, and then yearly or at the time of events. This study was approved by the Institutional Review Board of KNUCH (KNUCH_07–0033).

### Subjects

Among the 775 breast cancer patients who underwent NAC between January 2009 and December 2015, 109 female patients (median age 47 years; range 29–68 years) with an immunohistochemically-defined HR-positive, HER2–negative tumor were selected. The primary tumor features including clinical stage with tumor size and lymph node involvement, estrogen receptor (ER) status, progesterone receptor (PR) status, HER2 status, and Ki67 expression index are presented in Table 1. All patients (excluding two with cT4 who underwent additional T4 after AC4) received the planned regimens followed by curative surgical resection; one patient received fewer than the planned courses (6 out of 8 cycles) because of intolerance.

**Table 1:** Preoperative characteristics of 109 ER-positive/HER2-negative breast cancer patients who underwent neoadjuvant chemotherapy

	Number	Percent
Median age, years (range)	47.0 (29–68)	
Menopausal status		
Pre	75	68.8
post	34	31.2
Histology		
Ductal	103	94.5
Lobular	3	2.8
Metaplastic	2	1.8
mucinous	1	0.9
Tumor size (cT)^a^		
1	8	7.3
2	77	70.6
3	18	16.5
4	6	5.5
Lymph node involvement (cN)^a^		
0	8	7.3
1	46	42.2
2	45	41.3
3	10	9.2
Clinical stage (cS)^a^		
2A	10	9.2
2B	36	33.0
3A	48	44.0
3B	5	4.6
3C	10	9.2
ER, Allred score		
0–2	8	7.3
3–5	14	12.8
6–8	87	79.8
PR, Allred score		
0–2	13	11.9
3–5	20	18.3
6–8	76	69.7
Ki67 index, %		
< 14	54	49.5
14–100	55	50.5
Molecular subtype^b^		
Luminal A-like	48	44.0
luminal B-like	61	56.0
Regimens		
AC4T4^c^	103	94.5
AC4	4	3.7
TC4	2	1.8
Surgery		
BCS	36	33.0
mastectomy	73	67.0
SUVmax on ^18^F-FDG PET/CT at diagnosis		
SUVmax at breast (pSUVmax), mean + SD	9.19 ± 6.34	
< 9.55	66	60.6
≥ 9.55	43	39.4
SUVmax at axilla, mean + SD	6.14 ± 50.3	
ND^d^	23	21.1
x < 6.14	55	50.5
≥ 6.14	31	28.4

*Abbreviations*: *ER* Estrogen receptor, *PR* Progesterone receptor, *AC* Anthracycline + cyclophosphamide, *T* Taxane, *TC* Docetaxel + cyclophosphamide, *BCS* Breast conserving surgery, *PET* Positron emission tomography, *SUV* Standard uptake value, *ND* Not detected

^a^AJCC 8th edition

^b^Estimated by the results of immunohistochemical staining for ER, PR, HER2, and Ki67

^c^One patient received fewer than the planned number of courses (6 out of 8 cycles) because of intolerance

^d^Includes 8 cN0 cases

### Pathologic assessment

The tumor histology and biologic parameters were evaluated on both core-needle biopsy at initial diagnosis and the surgical specimen. Immunohistochemistry (IHC) was performed on formalin-fixed, paraffin-embedded tissue and ER and PR expression were scored according to the ASCO/College of American Pathologists (CAP) guidelines and graded by the Allred system [19]. Allred score is semi quantitative system that takes into consideration the proportion of positive cells (scored on a scale of 0–5) and staining intensity (scored on a scale of 0–3). The proportion and intensity were then summed to produce total scores of 0 or 2 through 8. A score of 0–2 was regarded as negative while 3–8 as positive. HER-2 positivity was defined as 3+ by IHC and/or by gene amplification using in situ hybridization (ISH). The Ki67 expression index was categorized with a cutoff of 14% [20]. Molecular subtype was estimated based on these four IHC results and/or HER2 ISH, as defined in a prior report [21]. Tumors were classified into 4 subtypes: luminal A (ER+ and/or PR+, HER2−); luminal B (ER+ and/or PR+, HER2+;ER+ and/or PR+, HER2-Ki 67+); basal (ER−, PR−, HER2−) and HER2/neu (ER−, PR−, HER2+). Histologic grade was determined using the modified Scarff-Bloom-Richardson grading system but was not calculated at baseline because the small-sized specimens acquired by core biopsy were insufficient to interpret mitosis count [22]. After surgery, pCR was defined as the absence of invasive cancer cells in both breast and axillary lymph nodes.

### PET imaging

^18^F-FDG PET/CT was performed at baseline before NAC and/or before surgery and all patients fasted for at least 6 h before ^18^F FDG administration, which was confirmed by serum glucose concentration (less than 150 mg/dl). All imaging studies were obtained with a hybrid PET/CT scanner and PET data were reconstructed iteratively according to the standard procedure described previously [23]. The SUV was defined as:

Tracer concentration [kBq/mL] / injected activity [kBq]/patient body weight [g].

The SUVmax on ^18^F-FDG-PET imaging was measured for both breast and axilla by two experienced nuclear medicine physicians, but only SUVmax of the primary breast tumor (pSUVmax) was used in analyzing the relationship with pCR and other clinical outcomes, as SUVmax of the axilla correlated with clinical N stage. We defined axillary lymph nodes as not detected (ND) in cases with clinically negative nodes or discordance with other images or pathological findings.

### Statistical analysis

Data are presented as numbers (%) or mean ± standard deviation unless otherwise stated. To evaluate the association of pSUVmax with variable parameters, subjects were divided into two groups based on the mean value of pSUVmax. Frequencies were compared using the chi-square test for categorical variables, and logistic regression models were used for identifying predictive factors for pCR among expected clinical and pathological variables including pSUVmax. Relapses were categorized as local, regional, and systemic recurrence, and invasive disease-free survival (IDFS) was calculated as the time between the date of diagnosis to the date of systemic recurrence and analyzed by the Kaplan-Meier method; the differences were assessed using the log-rank test, and each hazard ratio (HR) and 95% confidence interval (CI) was calculated using a cox-regression analysis. Receiver-operating characteristic (ROC) analysis was performed to determine the optimal cutoff value of pSUVmax in predicting pCR.

A *P* value of less than 0.05 was considered to be statistically significant. Analyses were conducted with IBM SPSS 22.0 (SPSS Inc., Chicago, IL, USA) and GraphPad Prism 7 (GraphPad Software, Inc., La Jolla, CA, USA).

## Results

### Patient characteristics

Among the 109 patients, most were node positive, ER, and PR positive (92.7, 92.7, and 88.1%, respectively), and 55 (50.5%) had a high Ki67 expression index (≥14%) at initial diagnosis, showing 44% luminal A-like subtype based on the study definition (Table 1). After NAC, approximately one-third received breast conserving surgery and pCR was observed in 8 patients (7.3%). The pathological stages in patients with residual tumor after NAC (*n* = 101) were as follows: 1A (*n* = 21, 19.3%), 1B (*n* = 7, 6.4%), 2A (*n* = 25, 22.9%), 2B (*n* = 20, 18.3%), 3A (*n* = 21, 19.3%), and 3C (*n* = 7, 6.4%). In the specimens from the 94 patients with residual breast tumor cells after surgery, the Ki67 expression index was lower than in their pretreatment tumors (*p* = 0.046).

### Relationship between pSUVmax and Clinicopathological parameters

**Fig. 1:**
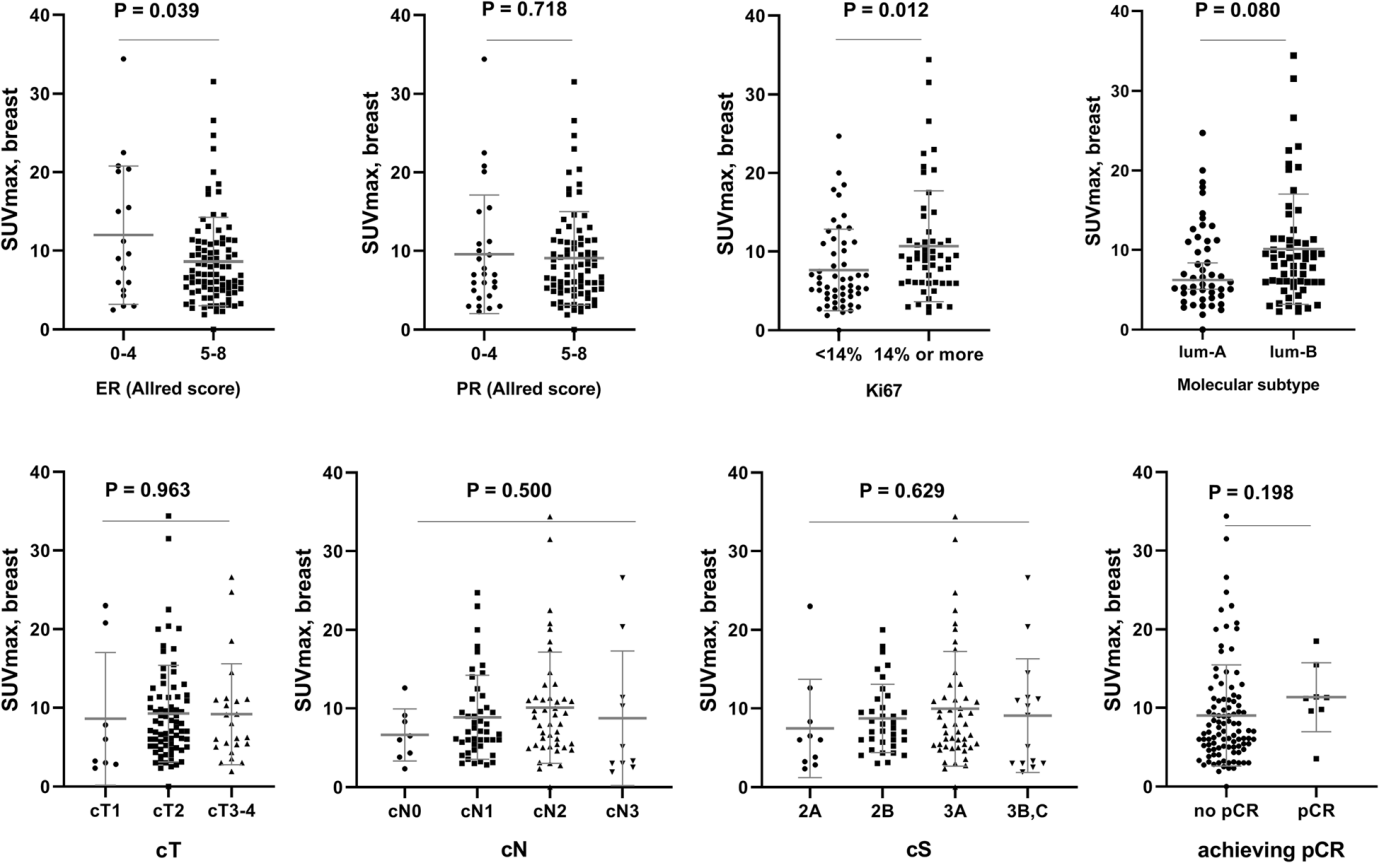
Comparisons of SUVmax of breast with pathological characteristics: (upper) *P* values are 0.039, 0.718, 0.012, and 0.080 for ER, PR, Ki67%, and molecular subtype, respectively. (lower) P values are 0.963, 0.500, 0.629, and 0.198 for cT, cN, cS, and pCR, respectively. Mean values of pSUVmax are indicated and the error bars represent the 95% confidence interval for the mean

**Fig. 2:**
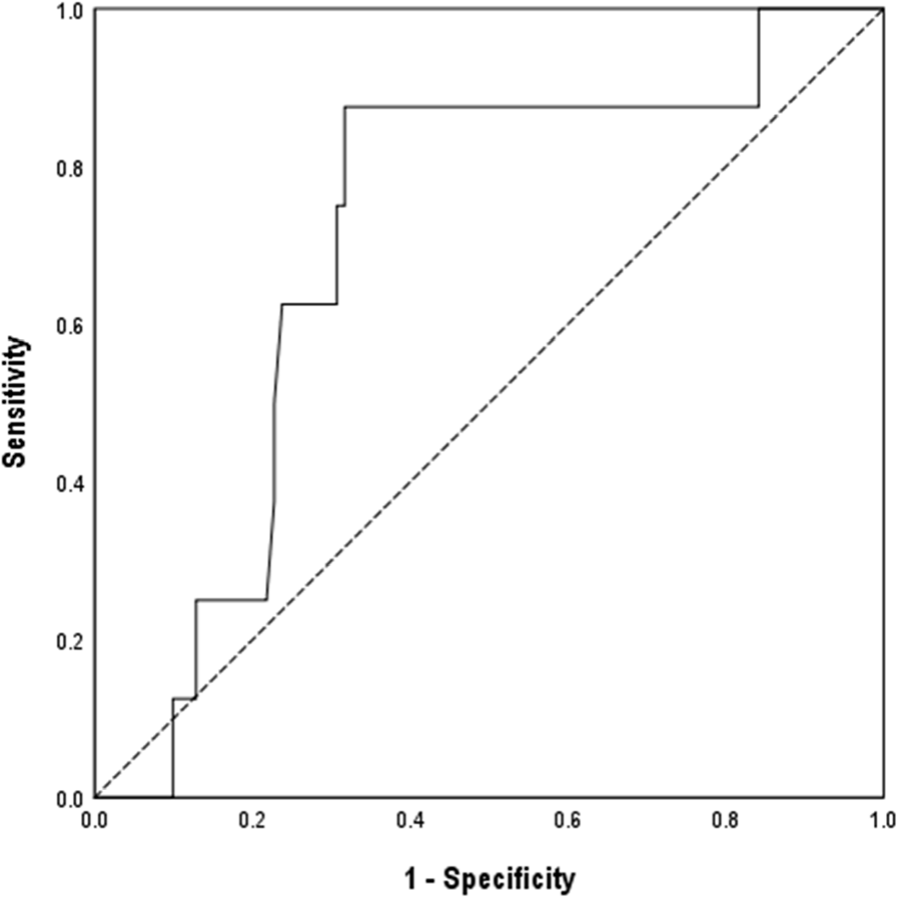
ROC curve of SUVmax of breast for predicting pCR after NAC: ROC curve demonstrating a pSUVmax of 9.55 as the optimal cutoff for predicting pCR (area under the curve: 0.703; standard error: 0.084), yielding a sensitivity of 87.5% and a specificity of 69.3%

The mean SUVmax of the breast and axilla tumors was 9.19 (range, 0–34) and 6.14 (range, 0–26), respectively (Table 1). The SUVmax of the axilla was significantly correlated with clinical N stage (cN) and thus excluded from further analysis. The mean pSUVmax was relatively higher in the luminal B-like subtype (10.11 vs. 8.03; *p* = 0.080), and high Ki67 expression groups (10.69 vs. 7.67; *p* = 0.012), but not significantly different according to tumor burden and clinical TNM stage (Fig. 1). A ROC curve demonstrated a pSUVmax of 9.55 as the optimal cutoff for predicting pCR (area under the curve: 0.703; standard error: 0.084), yielding a sensitivity of 87.5% and a specificity of 69.3% (Fig. 2) and 39 patients (35.8%) had a high pSUVmax (Table 2).

**Table 2:** Associations between pCR and clinical/pathologic parameters from logistic regression analysis

	Variables	N	pCR	Univariate analysis			Multivariate analysis		
				*P*	OR	95% CI	*P*	OR	95% CI
Age, years	< 50	66 (60.6)	5 (7.6)	0.817	1		0.656	1	
	≥50	43 (39.4)	3 (7.0)		0.915	0.207–4.043		1.231	0.266–8.164
Menopausal status	Pre	75 (68.8)	5 (6.7)		1				
	Post	34 (31.2)	3 (8.8)	0.690	1.355	0.305–6.027			
Clinical stage	2A	10 (9.2)	0	0.997	–	–	0.890	–	–
	2B	36 (33.0)	3 (37.5)						
	3A	48 (44.0)	4 (50.0)						
	3B,C	15 (13.8)	1 (12.5)						
Tumor size	1	8 (7.3)	0	0.996	1				
	2	77 (70.6)	6 (7.8)		–	–			
	3–4	24 (22)	2 (8.3)		–	–			
LN involvement	0	8 (7.3)	0	0.979	1				
	1	46 (42.2)	4 (8.7)		–	–			
	2	45 (41.3)	3 (6.7)		–	–			
	3	10 (9.2)	1 (10.0)		–	–			
ER, Allred score	0–4	18 (16.5)	3 (16.7)	0.114	1				
	5–8	91 (83.5)	5 (5.5)		0.291	0.63–1.346			
PR, Allred score	0–4	27 (24.8)	2 (25)	0.988	1				
	5–8	82 (75.2)	6 (75)		0.987	0.187–5.205			
Ki67 index, %	< 14	54 (49.5)	2 (3.7)	0.168	1				
	≥14	55 (50.5)	6 (10.9)		3.184	0.613–16.531			
Molecular subtype	Luminal A-like	75 (68.8)	5 (4.2)	0.274	1		0.294	1	
	Luminal B-like	34 (31.2)	3 (9.8)		2.509	0.483–13.032		2.570	0.441–14.973
Regimen	AC4 or TC	4 (3.7)	1 (25.0)	0.206	1				
	AC4T4	105 (96.3)	7 (6.7)		0.214	0.020–2.338			
pSUVmax	< 9.55	70 (64.2)	1 (1.4)	0.013	1		0.013	1	
	≥ 9.55	39 (35.8)	7 (17.9)		15.094	1.782–127.880		17.452	1.847–164.892

Numbers in parentheses are percentages. *Abbreviations*: *ER* Estrogen receptor; *PR* Progesterone receptor; *pSUVmax* SUVmax of primary breast tumor, *CR* Complete response, *AC* Anthracycline + cyclophosphamide, *T* Taxane, *TC* Docetaxel + cyclophosphamide, *BCS* Breast conserving surgery, *OR* Odds ratio, *CI* Confidence interval

Although no significant correlations were found between pCR and pretreatment clinical and pathological characteristics of HR-positive, HER2-negative breast cancer, the patients having tumors with a high pSUVmax (≥9.55) achieved more pCR compared to the low pSUVmax group (17.9% vs. 1.4%, *p* = 0.013) (Table 2). Furthermore, multivariate logistic regression analysis indicated that a high pSUVmax is an independent predictive marker of pCR to NAC (odds ratio [OR] = 17.452; 95% CI = 1.847–164.892; *p* = 0.013) (Table 2) when analyzed with age, clinical stage, and molecular subtype.

### Survival analysis

**Fig. 3:**
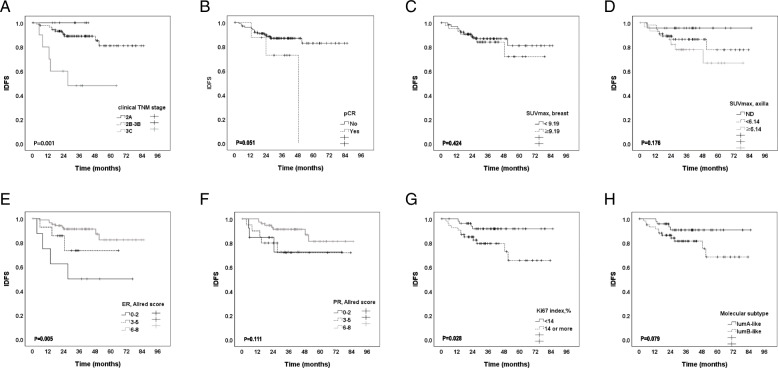
Invasive disease-free survival (IDFS) according to clinical stage (**a**), achieving pCR (**b**), pSUVmax (**c**), SUVmax of axilla (**d**), expressions of estrogen receptor (ER, **e**), progesterone receptor (PR, **f**) and Ki67 index (**g**), and molecular subtype (**h**)

During the follow-up period (median, 34.6 months; range, 0.5–85.3 months), eighteen patients (16.5%) experienced relapse (4 locoregional and 16 distant). Also, among 12 observed deaths, 11 were breast cancer-related (Table 3). Kaplan-Meier survival analysis demonstrated that advanced TNM stage, low ER expression, and high Ki67 were significantly associated with a worse IDFS (*p* = 0.001, 0.005, and 0.028, respectively) (Fig. 3). Multivariate survival analysis revealed that only clinical TNM stage was a prognostic factor for IDFS (HR and 95% CI, not calculated; *p* = 0.010; Table 4) However, pSUVmax and achievement of pCR were not associated with survival among the patients with HR-positive, HER2-negative breast cancer in the current study.

**Table 3:** Postoperative characteristics of 109 ER-positive/HER2-negative breast cancer patients who underwent neoadjuvant chemotherapy

	Number	Percent
Pathologic stage, postoperative^a^		
yp0 (pathologic CR)	8	7.3
yp1A	21	19.3
yp1B	7	6.4
yp2A	25	22.9
yp2B	20	18.3
yp3A	21	19.3
yp3B	0	0
yp3C	7	6.4
ER, Allred score^b^		
0–2	13	13.8
3–5	4	4.3
6–8	77	81.9
PR, Allred score^b^		
0–2	24	25.5
3–5	30	31.9
6–8	40	42.6
Ki67 index status^b^, %		
< 14	67	71.3
14–100	25	26.6
NE	2	2.1
Lymphovascular invasion^b^	37	39.4
Histological grade^b,c^		
G1	20	18.3
G2	56	51.3
G3	14	12.8
NE	19	17.4
Recurrence	18	16.5
Local	1	
Regional	1	
Regional ➔ distant	2	
Distant	14	
Death	12	11.0
breast cancer-related	11	10.1

*Abbreviations*: *CR* Complete response, *ER* Estrogen receptor, *PR* Progesterone receptor, *NE* Not evaluated

^a^AJCC 8th edition

^b^Evaluated among 94 patients who had residual tumors of the operated breast

^c^Modified Scarff-Bloom-Richardson grading system

**Table 4:** Survival analysis for invasive disease-free survival

Variables			Univariate analysis			Multivariate analysis		
			*P*	HR	95% CI	*P*	HR	95% CI
Age, years	≥50	< 40	0.330	1.629	0.611–4.341			
Menopausal status	Post	Pre	0.903	1.068	0.371–3.077			
Clinical stage	2B-3B	2A	0.007	–	–	0.010	–	–
	3C			–	–		–	–
ER, Allred score	5–8	0–4	0.003	0.221	0.082–0.593	0.216	0.425	0.109–1.651
PR, Allred score	5–8	0–4	0.129	0.456	0.166–1.258	0.597	0.671	0.153–2.945
Ki67 index, %	≥ 14	< 14	0.039	3.300	1.064–10.241	0.309	1.949	0.540–7.037
Molecular subtype	Luminal B-like	Luminal A-like	0.091	2.650	0.854–8.221			
Regimens	AC4T4	AC4 or TC4	0.599	21.513	–			
Surgery	Mastectomy	BCS	0.110	3.348	0.760–14.746			
pSUVmax	≥ 9.55	< 9.55	0.427	1.493	0.555–4.014	0.580	0.702	0.201–2.455
Pathologic CR	Yes	No	0.066	3.281	0.924–11.650	0.137	3.170	0.693–14.513

*Abbreviations*: *ER* Estrogen receptor, *PR* Progesterone receptor, *pSUVmax* SUVmax of primary breast tumor, *CR* Complete response, *AC* Anthracycline + cyclophosphamide, *T* Taxane, *TC* Docetaxel + cyclophosphamide, *BCS* Breast conserving surgery, *HR* Hazard ratio, *CI* Confidence interval

## Discussion

HR-positive, HER2-negative breast cancer is relatively common but less responsive to chemotherapy; in this setting, NAC is less likely to achieve pCR. Nonetheless, NAC can be frequently considered for patients with this subtype to obtain better surgical outcomes such as breast conservation. Therefore, good predictive markers in this subtype are needed for selecting chemotherapy before or after surgery.

Various factors have been proposed for the risk stratification of patients with breast cancer when considering adjuvant chemotherapy, such as tumor burden (T and N stage), histological grade, HR status, Ki67 expression index, and recently, gene signatures. However, these pathological predictors can be fully applied only after complete surgical excision and therefore have limited value in the neoadjuvant setting. On the other hand, ^18^F-FDG PET/CT can provide quantitative information about tumor glucose metabolism and be a valuable adjunct to conventional preoperative clinical assessment. In the current study, pSUVmax on PET images was relatively higher in cases with low ER expression and high Ki67 expression index and served as a potential predictive marker for pCR to NAC in patients with HR-positive,HER2-negative breast cancer subtypes, regardless of clinical stage or pathologic characteristics.

^18^F-FDG PET/CT using tumor glucose metabolism has been widely used for diagnosis, surveillance, or prognosis of various malignant tumors [14], but still has limited evidence of utility in breast cancer: the NCCN guidelines currently do not recommend its use in the staging of early breast cancer (www.nccn.org). Nonetheless, several studies have proven the association between SUV and breast cancer tumor burden, histological type, and aggressiveness [14, 24–26] and suggested that ^18^F-FDG PET/CT can predict treatment response in aggressive subtypes of breast cancer [27–29]. Furthermore, based on demonstration of the prognostic impact of pSUVmax among patients with various stages of breast cancer [23], we hypothesized its predictive role predicting treatment outcomes for specific treatment, particularly in the neoadjuvant setting. Although some prior studies demonstrated a change of SUV in response to chemotherapy as a predictive factor in aggressive breast cancer, such as the HER2 subtype [28], few studies have evaluated the predictive value of the pSUVmax in response to chemotherapy only in HR-positive, HER2-negative breast cancer patients. While patients with HR-positive breast cancer are believed to have a lower chance of pCR to NAC compared to those with HER2-positive and triple-negative subtypes [30, 31], the current findings suggest that ^18^F-FDG PET/CT may allow the identification of good responders to chemotherapy among patients with HR positive breast cancer; further studies for its use in breast cancer should be considered.

Meanwhile, achieving pCR is associated with better prognosis in patients with aggressive tumor subtypes and thus pCR has been accepted as a surrogate marker for long-term survival. However, this prognostic value was not found in a study involving HR-positive subtype tumors [11]. Similarly, in the current study, a high pCR rate in the group with high pSUVmax did not connote better survival. Instead, the pathologic stage of the residual tumors was significantly associated with survival when the patients achieving pCR were excluded (data not shown). These findings may indicate that HR-positive breast cancers are heterogeneous, having different levels of glucose metabolism, and the tumors with high pSUVmax may be more responsive but have a different clinical course compared to the others.

Currently, the HR-positive breast cancer is further subdivided into subtypes based on molecular expression: luminal A, B HER2-negative, and luminal B HER2-positive. The latter two subtypes have worse outcomes and need systemic chemotherapy even for early stage cancers [32]. Although gene expression profiling has become a more commonly used laboratory technique, it is still not broadly available as a validated diagnostic technique in most health care situations. Therefore, instead of DNA/RNA analysis, immunohistochemical analysis with 4 markers (ER, PR, HER2, and Ki67) have been used to define subtypes of breast cancer [21, 32, 33]. Thus, considering the limitations of immunohistochemical assay and specimens from core needle biopsy in the neoadjuvant setting, pSUVmax may be an alternative to molecular assays for identifying specific subtypes, potentially avoiding ineffective chemotherapies and permitting other treatment options such as neoadjuvant endocrine therapy or immediate surgery.

Meanwhile, the cutoff value requires further refinement in future studies, as the current values are too variable for use as a marker. Additionally, the PET technique enables metabolic pathway visualization of the increased glucose consumption in malignant tumors [15] and the activities of diverse glucose transporters such as glucose transporter I (GLUT-1) and intracellular glucose metabolic enzymes such as hexokinases have been shown to determine the level of FDG uptake in cancer tissue [34]. Therefore, further studies of the associations between these molecules and ^18^F-FDG PET/CT are warranted.

It is well known the incidence of pCR vary among breast cancer-intrinsic subtypes and the patients with HR-positive breast cancer show a low pCR rate compared with triple-negative or HER2-positive breast cancer patients [30, 31]. However, the small sample size and relatively lower incidence of pCR compared to that of other NAC studies limit definite conclusions. The lower incidence of pCR can be explained by the higher proportion of luminal A subtype in the study population. Nevertheless, despite the unproven role of PET scanning and its decreasing use in our region, this study may stimulate new insights into PET scanning. Moreover, the number of enrolled patients with HR-positive, HER2-negative early breast cancer is high compared to that of other studies of the role of PET in the neoadjuvant setting, and, to our knowledge, this study is the first to establish the role of initial pSUVmax as a noninvasive predictive marker of pCR to NAC.

## Conclusions

In this study, patients with HR-positive breast cancer generally have a low incidence of pCR to NAC vand therefore are infrequent candidates for NAC. However, the results of the current study suggest that PET imaging may be a good modality for selecting the initial therapeutic plan and possibly optimizing the chance of breast preservation in patients with HR-positive, HER2-negative type (especially luminal B-like type) breast cancer.

## Supplementary information

**Additional file 1.**

## Data Availability

The datasets generated or analysed during this study are not publicly available to protect the confidentiality of the subjects but are available from the corresponding author on reasonable request.

## Abbreviations

AC: Anthracycline + cyclophosphamide
CI: Confidence interval
ER: Estrogen receptor
^18^F-FDG PET/CT: Fluorine-18 fluorodeoxyglucose positron emission tomography
HER2: Human epidermal growth factor 2
HR: Hormone receptor
IDFS: Invasive disease-free survival
IHC: Immunohistochemistry
ISH: In situ hybridization; NAC: neoadjuvant chemotherapy
pCR: Pathologic complete response
PR: Progesterone receptor
pSUVmax: SUVmax of the primary breast tumor
ROC: Receiver operating characteristic
SUV: Standardized uptake value
T: Docetaxel
TC: Docetaxel + cyclophosphamide
