# Supplementary material for: Utility of 18F-FDG PET/CT for predicting pathologic complete response in hormone receptor-positive, HER2-negative breast cancer patients receiving neoadjuvant chemotherapy
Source: BMC Cancer. 2020 Nov 16;20:1106. doi: 10.1186/s12885-020-07505-w (PMC7667770; doi:10.1186/s12885-020-07505-w)
Supplement: Supplementary file 1 — Additional file 1. [file 12885_2020_7505_MOESM1_ESM.docx]

Supplementary Table 1

|  | Variables | N | pCR |  |  |
| --- | --- | --- | --- | --- | --- |
|  |  |  |  | Non-pCR | p-value |
| Age, years | <50 | 66 (60.6) | 5 (7.6) | 61(60.4) | 1.000 |
|  | ≥50 | 43 (39.4) | 3 (7.0) | 40(39.6) |  |
| Menopausal status | Pre | 75 (68.8) | 5 (6.7) | 70(69.3) | 0.703 |
|  | Post | 34 (31.2) | 3 (8.8) | 31(30.7) |  |
| Clinical stage | 2A | 10 (9.2) | 0 | 10(9.9) | 0.842 |
|  | 2B | 36 (33.0) | 3 (37.5) | 33(32.7) |  |
|  | 3A | 48 (44.0) | 4 (50.0) | 44(43.6) |  |
|  | 3B,C | 15 (13.8) | 1 (12.5) | 5(5) |  |
| Tumor size | 1 | 8 (7.3) | 0 | 8(7.9) | 0.708 |
|  | 2 | 77 (70.6) | 6 (7.8) | 71(70.3) |  |
|  | 3-4 | 24 (22) | 2 (8.3) | 22(21.8) |  |
| LN involvement | 0 | 8 (7.3) | 0 | 8(7.9) | 0.827 |
|  | 1 | 46 (42.2) | 4 (8.7) | 42(14.6) |  |
|  | 2 | 45 (41.3) | 3 (6.7) | 42(41.6) |  |
|  | 3 | 10 (9.2) | 1 (10.0) | 9(8.9) |  |
| ER, Allred score | 0-4 | 18 (16.5) | 3 (16.7) | 15(14.9) | 0.124 |
|  | 5-8 | 91 (83.5) | 5 (5.5) | 86(85.1) |  |
| PR, Allred score | 0-4 | 27 (24.8) | 2 (25) | 25(24.8) | 1.000 |
|  | 5-8 | 82 (75.2) | 6 (75) | 76(75..2) |  |
| Ki67 index, % | <14 | 54 (49.5) | 2 (3.7) | 52(51.5) | 0.271 |
|  | ≥14 | 55 (50.5) | 6 (10.9) | 49(48.5) |  |
| Molecular subtype | Luminal A-like | 48(60.6) | 2(12.5) | 46(64.4) | 0.462 |
|  | Luminal B-like | 61(39.4) | 6(87.5) | 55(35.6) |  |
| Regimen | AC4 or TC | 4 (3.7) | 1 (25.0) | 3(3.3) | 0.266 |
|  | AC4T4 | 105 (96.3) | 7 (6.7) | 98(97) |  |

Numbers in parentheses are percentages. **Abbreviations**: ER, estrogen receptor; PR, progesterone receptor; pSUVmax, SUVmax of primary breast tumor; CR, complete response; AC, anthracycline + cyclophosphamide; T, taxane; TC, docetaxel + cyclophosphamide; BCS, breast conserving surgery; OR, odds ratio; CI, confidence interval
